# Morphological Variations between Korean and Southwestern Japanese *Lilium leichtlinii* Hook. f.

**DOI:** 10.3390/plants11152016

**Published:** 2022-08-02

**Authors:** Ji-Young Kim, Jong-Kuk Na, Jong-Hwa Kim

**Affiliations:** 1 Department of Horticulture, Kangwon National University, Chuncheon 24341, Gangwon, Korea; multi-jjang@hanmail.net; 2 Department of Controlled Agriculture, Kangwon National University, Chuncheon 24341, Gangwon, Korea; 3 Oriental Bio-Herb Research Institute, Kangwon National University, Chuncheon 24341, Gangwon, Korea

**Keywords:** morphological traits, distribution, PCA, clustering

## Abstract

This study aimed to examine detailed morphological variations within *Lilium leichtlinii* Hook. f. For investigation, two groups, Korean *L. leichtlinii* (KR group) and southwestern Japanese broad-leaved *L. leichtlinii* (JSW group), were compared. In total, 52 morphological characteristics (45 quantitative and 7 qualitative traits) were examined in 59 lily accessions (30 KR and 29 JSW). Forty quantitative traits showed significant heterogeneity (*p* < 0.05) between JSW and KR accessions, and all seven color-related qualitative traits also exhibited differences. Student’s *t*-tests and principal component analysis (PCA) revealed that major quantitative morphological differences between the two groups included plant height, internode length, upper leaf size, and number of new bulbs. Cluster analysis of 36 morphological traits showed that the KR and JSW accessions belonged to two distinct groups. All together, these results indicate that KR and JSW groups are distal within *L. leichtlinii*, suggesting that the two groups could be considered different varieties.

## 1. Introduction

The name *Lilium leichtlinii* Hook. f. was initially given to Maximowicz’s citron-yellow lily by Joseph Dalton Hooker in 1867 [1]. On exactly the same day, Maximowicz’s orange lily was named as *L. pseudotigrinum* Carrier by Elie-Abel Carrière [2] because its morphology resembles that of *L. tigrinum*, also known as *L. lancifolium*. Additionally, this beautiful lily species has various other names [3,4,5,6,7,8,9], including *L. maximowiczii* Regel, *L. maximowiczii* var. *tigrinum* Regel, and *L. maximowiczii* var. *regelii* Elwes, which were given mostly on the basis of a few specimens without sufficient investigation of the variations [10]. In the modern era, however, the names, *L. leichtlinii* Hook. f. or *L. leichtlinii* var. *maximowiczii* (Regel) Baker are commonly used for all Maximowicz’s lilies.

*Lilium leichtlinii* is distributed widely in Northeast Asia, from the Ryukyu islands (26° N 128° E) to the Russian Far East (51° N 143° E), at altitudes of 5–2000 m. This wide distribution has precluded in-depth surveys, which would take tremendous effort and time but provide crucial information about infraspecific taxa of *L. leichtlinii*. One of infraspecific variation was reported in the ploidy of Korean *L. leichtlinii*. In Korea, there are two different ploidy *L. leichtlinii*, diploid and triploid plants, and the latter was reported bigger than the diploid plants in most morphological characteristics [11]. However, in Japan only diploid *L. leichtlinii* has been found and is called ‘Ko-oniyuri’, meaning ‘small tiger lily’ [12], because it is smaller than *L. lancifolium* Thunb. In Korea, *L. leichtlinii* is about 1.5 m in height and taller than *L. lancifolium* Thunb. in natural habitats [11,13,14]. This difference in plant height between Korean and southwestern Japanese *L. leichtlinii* could be a continuous variation or due to different environmental conditions since Korean *L. leichtlinii* is distributed in a cool temperate zone [15], while southwestern Japanese *L. leichtlinii* is distributed in a warm temperate region [16]. However, what caused such differences in plant height has not been investigated thoroughly. Shimizu [17] classified Japanese *L. leichtlinii* into two forms based on the leaf width: a narrow-leaf type (leaf width 0.4–0.5 mm) in the alpine regions in central Honshu and a wide-leaf type (leaf width 1–1.5 cm) in the southwestern coastal region. He recognized the latter as *L. maximowiczii* var. *regelii* Elwes. However, further in-depth morphological characteristics of the wide-leaf type of *L. maximowiczii* var. *regelii* Elwes were not investigated, leaving huge room to delve into.

We have long explored lilies in both Korea and southwestern Japan [11,18,19,20,21] and observed that southwestern Japanese *L. leichtlinii* is morphologically very different from Korean *L. leichtlinii*. Therefore, it was of interest to investigate whether the morphological differences observed in the survey are substantial enough to provide any evidence for potential variations within infraspecific taxa of *L. leichtlinii*. In this regard, this study was carried out to examine the morphological variations between two diploid *L. leichtlinii* groups, southwestern Japanese and Korean *L. leichtlinii*. We examined 45 quantitative morphological characteristics, 40 of which showed significant differences between the two groups. These findings are not only the first in-depth investigation of morphological variations between infraspecific taxa of *L. leichtlinii* but also indicate that two groups could be considered as two different varieties.

## 2. Materials and Methods

### 2.1. Materials

**Figure 1:**
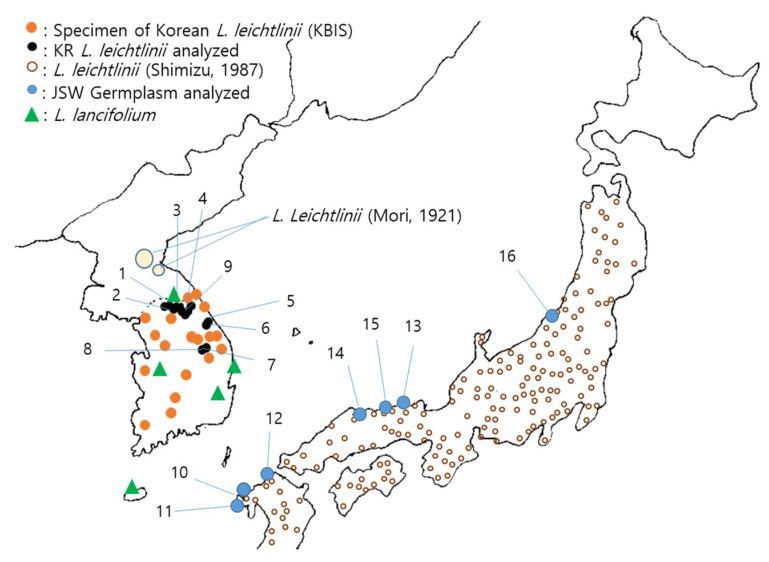
Distribution maps and collection sites of *L. leichtlinii* accessions in Korea and Japan (numbers 1–16 refer to numbers in Table 1). Distribution maps of *L. leichtlinii* are drawn based on the present study, reports by Mori [24] and Shimizu [12], and information on specimen distribution from KBIS [25].

Korean *L. leichtlinii* accessions used in this study were collected by the authors [11] and maintained in the germplasm field at Kangwon National University (KNU), Chuncheon, Korea. Japanese *L. leichtlinii* seeds were collected from seven regions of the southwestern coast of Japan, from Hirado in the south to Niigata in the northwest, in 2005 (Table 1; Figure 1). Adult plants grown from the seeds were maintained at the same KNU germplasm field with Korean *L. leichtlinii*. Korean and southwestern Japanese *L. leichtlinii* accessions were designated as KR and JSW, respectively.

To obtain morphological data from JSW plants in their natural habitats, we examined nine accessions, two from Hirado, five from Tottori, and two from Ishiji, Japan, during the flowering period. In addition, 20 more JSW accessions were investigated for 52 morphological characteristics (45 quantitative and 7 qualitative) at the KNU germplasm field. Thirty diploid KR accessions were collected from five districts (Hwacheon, Cheolwon, Gangneung, Wonju, and Pyungchang) in Kangwon province, Korea, and used for the analysis (Table 1). Ploidy level of JSW accessions was verified by a flow cytometer as described previously [11]. Triploid KR *L. leichtlinii* were not included in this study, because they are more robust and taller than diploids and because they are not found in Japan.

### 2.2. Morphological Analysis

Morphological investigation was based on the lily trait survey table reported by Asano [22]. Specific characteristics of the spots, nectar furrow, underground parts, and upper and middle leaves were also included in the survey (Table 2). The plant height was measured to the last bud during the flowering period, and the peduncle length was measured from the last leaf to the first pedicel. The length and thickness of the pedicel was measured for only the first flower. The number of leaves was calculated by including the number of nodes without leaves at the lower parts of plants. The bract was measured from the bract of the pedicel of the first flower. The number of spots, the length of the longest spot, and the length of the nectar furrow were measured on the inner tepal. The five leaves from the top were denoted as the first to fifth leaves, in order. The color of plant parts or organs was measured based on the Royal Horticultural Society color chart [23]. For assessment of characteristics of fruits and underground parts (numbers 37–45 in Table 2), 20 KR and 20 JSW plants were randomly selected.

### 2.3. Statistical Analysis

All applied analyses were performed using tools embedded in a standalone Jamovi statistical software (version 2.2.5.0) obtained from the Jamovi website (https://www.jamovi.org). Student’s *t*-test was performed and significance was indicated by the *p* value (Table 2). Sample normality and homogeneity were examined for traits 1–36 in Table 2 using the Levene’s homogeneity variance and Shapiro–Wilk normality test tools, which resulted in most samples being of equal variances and distributed normally, with some exceptions. Principal component analysis (PCA) and cluster analysis were carried out for the traits 1–36 in Table 2. Briefly, 9 traits, traits 37–45 in Table 2, were excluded from the analyses because their sample sizes were smaller than those of other traits. PCA was performed with the varimax rotation method. Cluster analysis was carried out using the similarity of Euclidean distance tool. Linear discriminant analysis was performed using a snowcluster tool.

## 3. Results

### 3.1. Characteristics of Korean and Japanese L. leichtlinii Habitats

In Korea, KR is distributed in the inland of three provinces: Gyeonggi, Gangwon, and Chungcheongbukdo [11], located in a cool temperate zone [26]. In these regions, winter temperature drops below –20 °C and even in summer it is lower by 2–7 °C than the mean summer temperature of South Korea. On the other hand, JSW accessions are distributed widely in Japanese southwest coastal regions, a warm climate temperate zone [16], where the lowest average temperature is 5 °C (https://ja.weatherspark.com/y/142694/Hirado-137). 

Most KR plants inhabited sedimentary or sandy soil near rivers or small streams, although some inhabited alpine areas near mountain ridges or treeless open areas covered with grass. Most JSW plants were found in coastal sand dunes or low hills with well-draining sandy or humus soils.

### 3.2. Morphological Characteristics

**Figure 2:**
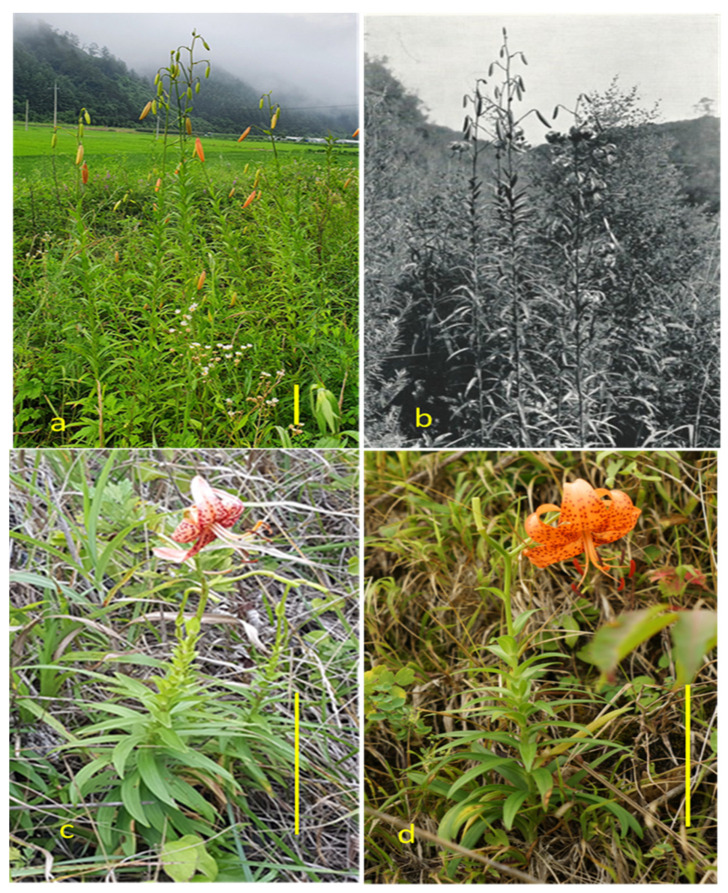
*L. leichtlinii* morphology in native habitats in Korea and Japan. (**a**). Wonju, Kangwon-do, Korea. (**b**). North Korea [27]. (**c**). Ishiji, Niigata, Japan. (**d**). Ikistuki, Hirado, Japan. Bars indicate 20 cm.

We examined 45 quantitative morphological traits for JSW and KR accessions (Table 2) and found significant differences in 40 of them between the two groups (Table 2). Figure 2a and 2b show photographs of KR *L. leichtlinii* in two different habitats taken at different times: the former in Wonju, Gangwon, Korea, in 2021 and the latter in Sepo, Gangwon, North Korea, by Willson [27]. The plants in the two images have very similar morphology and plant heights exceeding 150 cm (Figure 2a). By contrast, plant heights of JSW *L. leichtlinii* in coastal habitats in Niigata (Figure 2c) and Hirado (Figure 2d) were less than 60 cm. 

**Figure 3:**
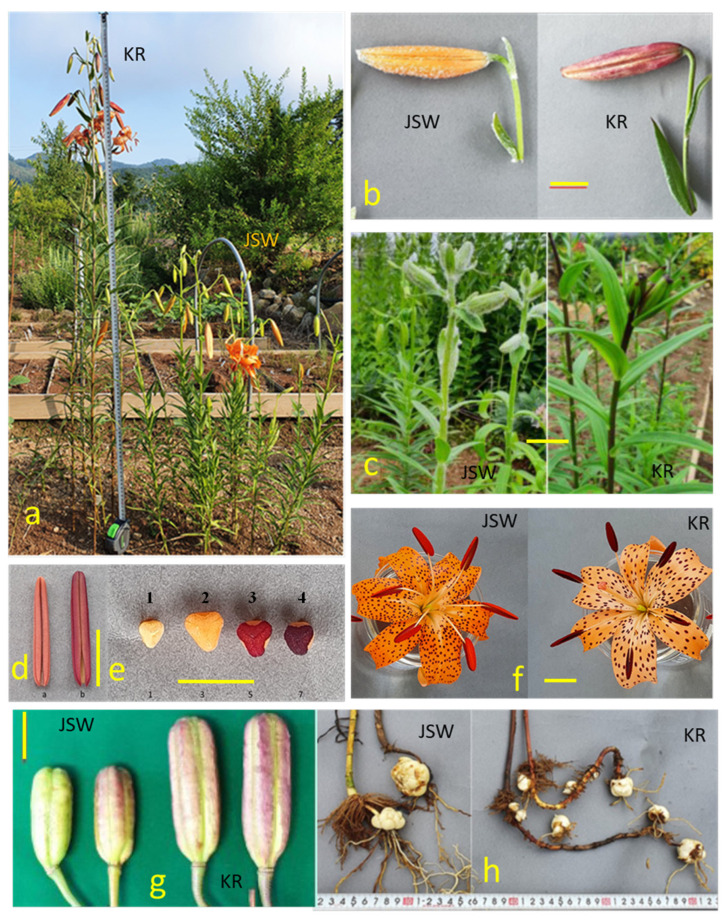
Comparison of KR and JSW *L. leichtlinii*. (**a**). Plant height of representative KR and JSW *L. leichtlinii* in germplasm field. (**b**). Bud morphology. (**c**). Stem color and hairs on the shoots and flower bud. (**d**). Anther color of JSW (**a**) and KR (**b**) plants. (**e**). Stigma color of JSW (#1) and KR plants (#2-4). (**f**). Flower morphology. (**g**). Fruit size. (**h**). Size of fully developed stolons. Scale bar of (**b**,**c**,**f**) = 2 cm; (**d**,**e**,**g**) = 1 cm.

Further detailed morphological traits of KR and JSW were examined and compared in the germplasm field (Figure 3). KR was significantly taller than JSW (Figure 3a). Flower and floral organs showed different colors (Figure 3b,d–f; Table 3). Fruit size of JSW was significantly smaller than that of KR (Figure 3g). All JSW plants had green stems, but KR plants had brown to dark brown stems (Figure 3c). Additionally, JSW plants had large numbers of differentiated and clustered leaves on the upper parts of their short stems (Figure 2c,d; Figure 3a). Pubescence appeared on the stem after sprouting in early spring, but most of the hairs disappeared during the flowering period. In some plants, however, numerous hairs remained on the upper stem during the flowering period (Figure 3c). About 50% of JSW accessions were hairy, whereas only a few hairy accessions were found in the KR group (Figure 3b). Stolons of KR plants were significantly longer than those of JSW plants because they extended for much longer distances before sprouting (Table 2; Figure 3h). JSW plants had only one maternal bulb, whereas KR plants developed three new bulbils on average, which were smaller than the mother bulb (Table 2; Figure 3h).

### 3.3. PCA

In a PCA analysis using 36 traits, the first three principal components explained 60.0% of the total variation (Supplementary Table S1). The traits with high loading values (marked in bold in Table S1) in the first principal component, PC1, were related to the first five leaves from the top. High loading values (marked in bold in Table S1) observed in PC2 were related to floral organs, including the length of the outer tepal (LOT), length of the inner tepal (LIT), diameter of the stigma (DOSt), and length of the filament (LOF). Representative traits from PC3 were related to plant size, including plant height (PLH), length to first flower (LT1F), length to last leaf (LTLL), and the length of the internode (LIN).

**Figure 4:**
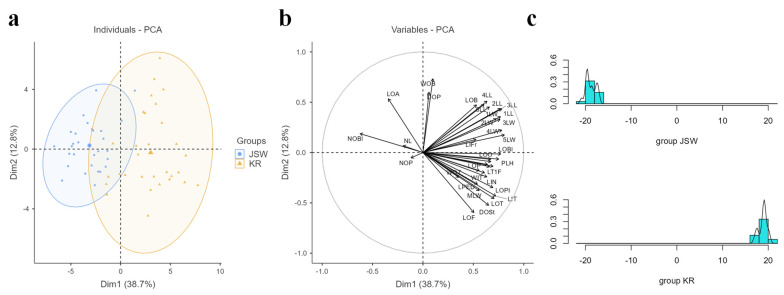
PCA plots and linear discriminant analysis. (**a**). PCA individual plot based on traits 1–36 traits in Table S1. (**b**). PCA variable plot. (**c**). Linear discriminant analysis showed distinct separation between JSW and KR *L. leichtlinii*.

As expected from the significant differences observed in 40 quantitative morphological traits between the two groups (Table 2), the PCA individual plot chart also showed JSW and KR accessions as two distinct groups (Figure 4a). Among 36 traits, 32 traits were positively correlated and 4 traits (NOP, NL, NOBI, and LOA) were negatively correlated with PC1 (Figure 4b), whereas 17 traits were positively correlated and 19 traits were negatively correlated with PC2 (Figure 4b). Traits LOT, LIT, LOF, and DOSt, with high loading values in PC1, showed close relationships. Traits 3LL, 2LL, 4LL, 1LL, and 5LL, with high loading values in PC2, also showed close relationships and were positively correlated to PC1 and PC2 (Figure 4b). Among the four traits negatively correlated with PC1, three traits (NL, NOBI, and LOA) were positively correlated with PC2. Based on the PCA plots, NOBl and LOA are likely major factors that affect JSW morphology, whereas PLH, LOBl, and LOO are major factors influencing KR morphology (Figure 4a,b).

Linear discriminant analysis produced results that were consistent with the PCA (Figure 4c). These results indicate that various traits could be used to distinguish between the JSW and KR groups.

### 3.4. Cluster Analysis

**Figure 5:**
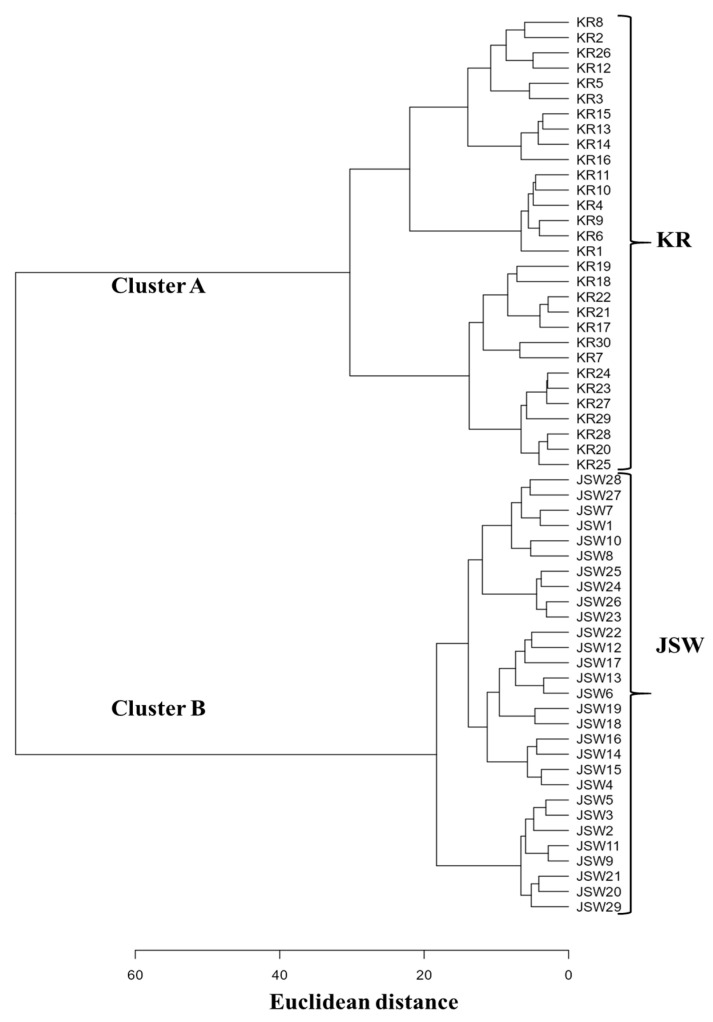
Hierarchical cluster analysis based on 36 morphological characteristics of two *L. leichtlinii* groups: KR and JSW. KR and JSW accessions showed distinct separation from each other, as KR *L. leichtlinii* accessions grouped into cluster A and JSW accessions into cluster B.

Cluster analysis of 59 *L. leichtlinii* accessions generated two distinct clusters, designated cluster A and B. All KR accessions were grouped into cluster A, whereas all JSW accessions formed cluster B (Figure 5). This result implies that JSW and KR, to some extent, are different groups within *L. leichtlinii* and provides evidence to support the separation of JSW and KR into different varieties.

## 4. Discussion

### 4.1. Morphological Characteristics in the Germplasm Field and in Natural Habitats

Most characteristics of the JSW accessions were similar in the germplasm field and in native habitats, but changes in plant growth and lengths of the internode and the stolon were observed. JSW plants showed elongated lower internodes in native grassy habitats but not in the germplasm field, likely due to the absence of grasses. JSW plants in natural habitats were taller and had slightly longer stolons compared to plants in the germplasm field. The upper leaves of JSW plants were extremely short in their native habitats (Figure 2c) but not in the germplasm field. Both JSW and KR plants grew better in natural habitats than in the germplasm field. Other quantitative traits did not differ significantly between plants growing in natural habitats and those grown in the germplasm field.

The short height of JSW plants in natural habitats might be an adaptation resulting from long-term exposure to strong sea wind [28]. To some degree, however, the height of JSW also is influenced by soil and surrounding conditions. Many JSW plants were less than 40 cm in height in rocky crevices along the seaside, but plants in fertile soil with partial shade grew up to 1.0 m. KR plants formed a widespread ramet in their natural habitat. KR plants with 1 or 2 flowers grew up to 1.0 m in height, but individuals with about 10 flowers reached more than 2.0 m in height, even within the same ramet. In general, lily plant height is proportional to the number of flowers, but only a few JSW individuals in natural habitats grew more than 1.0 m tall, even when they had a large number of flowers. In the germplasm field in inland Korea, only a few JSW accessions grew up to 1.0 m in height, with JSW plants exhibiting a mean height of 64 cm, similar to the heights of JSW plants in their natural habitats, supporting the idea that shorter height in JSW plants is an adaptation to harsh environments.

### 4.2. Morphological Differences between KR and JSW

Elwes [6] reported that there are different varieties within *L. leichtlinii* based on a few specimens. However, no in-depth morphological variations of *L. leichtlinii* have been described. In this study, we examined the morphological characteristics of two *L. leichtlinii* groups, KR and JSW, to examine whether there are variations within *L. leichtlinii*.

The height of JSW plants was significantly shorter than KR plants, which was one of key differences between two groups. The dwarf stature of JSW plants is noted in previous reports [17]. Shimizu [17] reported that JSW plants with wide leaves were *L. maximowiczii* var. *regelii* Elwes, known as a dwarf variety [6]. In addition, an illustration in Shohonzusetsu showed JSW plants characterized by small stature and very short internodes [29]. A more severe dwarf type of JSW plant was recently introduced as ‘Dangogooniyuri’, meaning ‘very small lily’ in Japanese [28].

We surveyed JSW distribution in Japan and found that JSW distributed only in the west coast region but not inland, including alpine regions in Tottori and Niigata prefectures. In alpine regions, including the wetlands of the Nagano ‘Hakuba’ (1400 m) and Tottori ‘Daisan’ (1500 m) areas, only the narrow-leaved *L. leichtlinii* was found. Additionally, the JSW-type *L. leichtlinii* was not found in Korea, including the Korean coastal region [11,19]. Based on these observations, JSW and the narrow-leaved *L. leichtlinii* likely do not represent continuous variation in the same group, as Shimizu [17] suggested. Intriguingly, however, KR and Japanese alpine *L. leichtlinii* show similarities in some traits, including plant height, internode length, and stem color. Some alpine *L. leichtlinii* plants were almost identical to KR plants in the preliminary survey, suggesting that they could represent continuous variation in the same group.

Closely related species can share ecological similarity [30], but JSW does not share ecological features with Japanese alpine *L. leichtlinii*, although the distance of their habitats is very proximal around 10–30 km. Our cluster analysis revealed that JSW is a distinct group from KR (Figure 5). Differences between KR and JSW were observed not only in quantitative traits, including those of fruit and underground parts (traits 37–45 in Table 2), but also in qualitative traits (Table 3). Taken together, these findings support the idea that JSW and KR could be considered as different varieties.

## 5. Conclusions

In this study, we examined morphological variations between two groups of *L. leichtlinii*, JSW and KR, and found that they were significantly different. The variations were obvious enough that the two groups could be distinguished by morphological traits, even without histological or genomic analysis. Among quantitative morphological variations, four traits were the most reliable for distinguishing between the two groups: (1) plant height, (2) internode length, (3) leaf density, and (4) size of leaves at the upper part of the plant. These findings are crucial evidence in determining that KR and JSW *L. leichtlinii* are different varieties. To obtain more detailed information about the infraspecific taxa of *L. leichtlinii*, studies would need to include populations from China and the Russian Far East.

## Figures and Tables

**Table 1:** Accessions and localities of Korean (KR) and southwestern Japanese (JSW) *Lilium leichtlinii* populations used in this study. All accessions used in this study were diploid.

No.	Group	Accession		Origin (GPS)	Altitude	Habitat
		Name *	Number **			
1	KR	Kimhwa (3)	IT283155	Mahyunri, Keunnamyeon, Cheolwongun, Gangwon, Korea (N38°16′22″, E127°31′43″)	278	Riverside
2	KR	Kimhwa (2)	IT283154	Mahyunri, Keunnammyeon, Cheolwongun, Gangwon, Korea (N38°15′18″, E127°34′51″)	260	Riverside
3	KR	Mahyun (1)	IT 283150	Mahyeonri, Sangseomyeon, Hwacheongun, Gangwon, Korea (N38°14′13″, E127°36′06″)	384	Streamside
4	KR	Hwacheon (2)	IT 283149	Mahyeon-ri, Sangseomyeon, Hwacheongun, Gangwon, Korea (N38°14′12″, E127°36′04″)	388	Riverside
5	KR	Amban (1)	IT 283132	Daegi-ri, Wangsanmyeon, Gangneung, Gangwon, Korea (N37°43′33″, E128°07′44″)	733	Mountain slope
6	KR	Hoengge (6)	IT283128	Hoenggyeri, Doammyeon, Pyeongchanggun, Gangwon, Korea (N37°36′53″, E128°43′33″)	776	Mountain slope
7	KR	Hwangdun (2)	GWL18055	Songgye-ri, Sillimmyeon, Wonj, Gangwon, Korea (N37°14′52″, E128°09′25″)	325	Streamside
8	KR	Jucheon (6)	IT 283158	Songgye-ri, Sillimmyeon, Wonj, Gangwon, Korea (N37°16′59″, E128°12′06″)	320	Streamside
9	KR	Sanyang (7)	GWL18056	Sanyang-Ri, Sangseo-myeon, Hwacheongun, Gangwon, Korea (N38°14′25″, E127°37′34″)	355	Streamside
10	JSW	Hirado (2)	GWL99089	Hirado, Nagasaki, Japan (N33°21′34″, E129°34′06″)	10	Seaside
11	JSW	Ikitsuki (1)	GWL99087	Ikitsuki, Hirado, Nagasaki, Japan (N33°22′05″, E129°26′03″)	10	Seaside
12	JSW	Tottori (9)	GWL00101	Tottori, Tottori, Japan (N35°31′31″, E134°06′35″)	15	Seaside
13	JSW	Matsue (4)	GWL00102	Matsue, Tottori, Japan (N35°34′17″, E133°13′14″)	15	Seaside
14	JSW	Nagasaki (2)	GWL00103	Nagatabira, Saga, Japan (N33°21′26″, E129°36′38″)	7	Seaside
15	JSW	Aoya (9)	GWL01104	Aoya, Tottori, Japan (N35°31′54″, E134°00′16″)	25	Sea cliff
16	JSW	Ishiji (2)	GWL01105	Niigata, Niigata, Japan (N37°31′04″, E138°39′19″)	8	Sea cliff

* Numbers in parentheses indicate the number of individuals used in this study. ** IT serial numbers are the accession numbers for registration in RDA Genebank Korea. GWL indicates Gangwon lily germplasm in Kangwon National University, Gangwon province, Korea.

**Table 2:** Summary of morphological characteristics of JSW and KR groups of *L. leichtlinii*.

No.	Characteristic (abb.) *			JSW (29)			KR (30)		Student’s *t*-Test
		Unit	Min	Mean ± SD	Max	Min	Mean ± SD	Max	*p* Value ***
1	Plant height (PLH)	cm	29	64.3 ± 16.6	105	102	131.5 ± 25.2	191	<0.001
2	Length to first flower (LT1F)	cm	33	56.9 ± 18.1	100	88	110.2 ± 19.6	156	<0.001
3	Length to last leaf (LTLL)	cm	27.5	49.9 ± 17.7	94	76	100.2 ± 19.6	146	<0.001
4	Length of inflorescence (LIFr)	cm	5	16.1 ± 6.5	30	14	21.1 ± 9.7	41	<0.670
5	Length of peduncle (LPED)	cm	4	6.9 ± 2.1	13	5	10.1 ± 2.5	15	<0.001
6	No. of leaves (NL)	cm	44	69.9 ± 22.4	130	37	56.1 ± 17.3	105	<0.007
7	Length of internode (LIN)	cm	0.45	0.87 ± 0.3	1.78	1.33	1.84 ± 0.4	2.51	<0.001
8	First leaf length (1LL)	cm	2.5	4.3 ± 1.3	8	2.5	6.4 ± 1.8	9.5	<0.001
9	First leaf width (1LW)	cm	0.7	1.3 ± 0.3	2	1	1.6 ± 0.3	2	<0.005
10	Second leaf length (2LL)	cm	2.5	4.8 ± 1.4	9	3	6.9 ± 1.9	12	<0.001
11	Second leaf width (2LW)	cm	0.7	1.3 ± 0.3	1.9	1	1.6 ± 0.3	2.2	<0.001
12	Third leaf length (3LL)	cm	2.8	5.31 ± 1.51	10	3	7.77 ± 2.02	11.5	<0.001
13	Third leaf width (3LW)	cm	0.7	1.22 ± 0.21	1.7	1	1.51 ± 0.25	2.2	<0.001
14	Fourth leaf length (4LL)	cm	3	5.77 ± 1.54	10	1.5	7.94 ± 2.17	13	<0.001
15	Fourth leaf width (4LW)	cm	0.7	1.17 ± 0.21	1.6	1	1.5 ± 0.24	2.2	<0.001
16	Fifth leaf length (5LL)	cm	3.2	6.26 ± 1.53	10	1.6	8.34 ± 2.22	12	<0.001
17	Fifth leaf width (5LW)	cm	0.7	1.12 ± 0.21	1.6	1	1.47 ± 0.21	1.9	<0.001
18	Middle leaf length (MLL)	cm	6.7	9.69 ± 1.45	13.1	10.9	12.3 ± 1.18	14.9	<0.001
19	Middle leaf width (MLW)	cm	0.6	0.88 ± 0.17	1.24	0.6	1.18 ± 0.22	1.68	<0.001
20	Length of pedicel (LOP)	cm	3	7.01 ± 2.16	12	6.2	8.7 ± 2.01	14	<0.003
21	Diameter of pedicel (DOP)	cm	0.24	0.31 ± 0.03	0.4	0.25	0.31 ± 0.03	0.4	<0.926
22	Length of bract (LOB)	cm	2	3.33 ± 0.70	5	2	4.39 ± 1.02	8	<0.001
23	Width of bract (WOB)	cm	0.8	1.51 ± 0.39	2.3	0.9	1.50 ± 0.36	2.5	<0.920
24	Length of outer tepal (LOT)	cm	5.4	7.9 ± 2.16	10	7	9.2 ± 1.29	13	<0.001
25	Width of outer tepal (WOT)	cm	1.3	1.86 ± 0.20	2.2	1.2	1.9 ± 0.27	2.4	<0.536
26	Length of inner tepal (LIT)	cm	5.5	8.06 ± 0.93	10	6.9	9.36 ± 1.34	13.6	<0.001
27	Width of inner tepal (WIT)	cm	1.9	2.49 ± 0.36	3	2	2.81 ± 0.36	3.9	<0.002
28	No. of blotches (NOBl)		52	136.31 ± 25.2	200	62	93.5 ± 16.05	140	<0.001
29	Length of blotch (LOBl)	mm	1	1.59 ± 0.71	4	1.2	3.04 ± 1.02	4.5	<0.001
30	Length of nectar furrow (LON)	cm	1.5	2.04 ± 0.31	2.7	1.6	2.43 ± 0.29	3	<0.001
31	Length of pistil (LOPI)	cm	5	5.87 ± 0.58	7	5.5	6.52 ± 0.58	7.8	<0.001
32	Length of ovary (LOO)	cm	1.4	1.79 ± 0.26	2.5	1.6	2.23 ± 0.36	2.7	<0.001
33	Diameter of stigma (DOSt)	cm	0.25	0.29 ± 0.02	0.4	0.25	0.38 ± 0.08	0.5	<0.001
34	Length of filament (LOF)	cm	3.5	5.21 ± 0.84	7	4	5.7 ± 0.71	6.9	<0.017
35	Length of anther (LOA)	cm	1.5	2.21 ± 0.39	3	1.3	1.9 ± 0.49	3	<0.027
36	No. of papillae (NOP)		0	0.34 ± 1.07	4	0	0.6 ± 1.37	6	<0.487
37 **	Fruit length	cm	3.7	4.26 ± 0.38	4.8	4.1	5.22 ± 0.63	6.2	<0.001
38 **	Fruit diameter	cm	1.6	1.74 ± 0.11	1.9	2	2.32 ± 0.21	2.7	<0.001
39 **	No. of seeds per capsule		186	210 ± 24.7	250	184	258 ± 50.6	281	<0.001
40 **	Mean seed length	mm	5.8	6.46 ± 0.37	7.9	6.3	7.63 ± 0.47	7.9	<0.001
41 **	Stolon length	cm	4	5.14 ± 0.89	7.5	25	27.7 ± 2.88	33	<0.001
42 **	No. of stolon nodes		2	2.6 ± 0.89	4	7	9.17 ± 1.33	11	<0.001
43 **	No. of bulblets		1	1 ± 0	1	2	4.17 ± 1.33	6	<0.001
44 **	Bulb width	cm	2.5	3.34 ± 0.74	4.5	3	3.48 ± 0.37	4.1	<0.001
45 **	Bulb height	cm	2	2.26 ± 0.25	2.5	2.3	2.53 ± 0.31	3.1	<0.001

* Traits 8–17 are characteristics of the first through fifth leaves starting from the uppermost leaf at the stem (N = 59). ** Traits 37–45 were not included in PCA and cluster analysis because small numbers of samples were examined. *** Levene’s test was performed and *p*-value less than 0.05 suggests a violation of the assumption of equal variances.

**Table 3:** Comparison of qualitative traits between JSW and KR *L. leichtlinii* (N = 59).

Trait	JSW	KR
Anther color	Greyed red 182B	Greyed purple 187A
Bud color	Orange 28B	Red 46A
Blotch color	Greyed red 178A	Greyed purple 187A
Pollen color	Greyed red 34A	Greyed purple 187B
Tepal color	Orange 28B, C	Orange red 32D to Orange 28C
Stigma color	Orange 28D	Orange 27A to Greyed purple 185A
Stem color	Yellow–green 145B	Yellow–green 145B to Greyed purple 183A

## Data Availability

Not applicable.

## Supplementary Materials

The following supporting information can be downloaded at: https://www.mdpi.com/article/10.3390/plants11152016/s1, Table S1: Matrix of coefficient vectors and variance proportion from the first three principal component axes based on 36 morphological traits of 59 *L. leichtlinii* accessions (30 KR and 29 JSW).

## References

[1] Hooker J.D. (1867). *Lilium leichtlinii* Max Leichtlin’s lily. Curtis’s Bot. Mag..

[2] Carriere E.A. (1867). *Lilium* *pseudotigrinum*. Rev. Hortic..

[3] Baker J.G. (1871). *Lilium leichtlinii* var. *maximowiczii*, Gardeners. Gard. Chron..

[4] Baker J.G. (1873). Baker’s classified list of all known lilies. J. Roy. Hort. Soc..

[5] Compton J.A. (2021). *Lilium leichtlinii* subsp. *maximowiczii* (Regel) J.Compton (Liliaceae): A new combination for Maximowicz’s orange lily. PhytoKeys.

[6] Elwes H.J. (1879). A Monograph of the Genus Lilium.

[7] Nicholson G. (1887). *Lilium leichtlinii* var. *tigrinum* (Regel) G. Nicholson. III Dict. Gard..

[8] Regel E.A. (1868). *Lilium* *maximowiczii*. Gartenflora.

[9] Regel E.A. (1870). *Lilium maximowiczii* var. *tigrinum*. Gartenflora.

[10] Mckenney J. (2011). Some thoughts on the intraspecific nomenclature of lilies. Lilies and Related Plants. J. R. Hort. Soc..

[11] Kim J.H., Truong N.X., Song Y.S., Kim N.S. (2016). Natural triploid *Lilium leichtlinii* var. *maximowiczii* populations in Korea. Plant Species Biol..

[12] Shimizu M. (1987). The Lilies of Japan.

[13] Makino T., Hara H., Tuyama T., Fumio M. (1961). Makino’s New Illustrated Flora of Japan.

[14] Lee Y.N. (2006). New Flora of Korea.

[15] Hayashi K., Kawano S. (2000). Molecular systematics of *Lilium* and allied genera (Liliaceae): Phylogenetic relationships among *Lilium* and related genera based on the *rbcL* and *matK* gene sequence data. Plant Species Biol..

[16] Hämet-Ahti L., Ahti T., Koponen T. (1974). A scheme of vegetation zones for Japan and adjacent regions. Ann. Bot. Fenn..

[17] Shimizu M. (1971). The Lilies of Japan.

[18] Kim J., Jang W., Kyung H., Yuan Z., Davaasuren Y., Sim E., Lee J., Choi Y., Hiramatsu M., Kim K. (2006). A Principal Component Analysis for the Morphological Characters of Diploid and Triploid Populations of *Lilium lancifolium* in Korea. Korean J. Plant Res..

[19] Kim J., Kyung H., Choi Y., Lee J., Hiramatsu M., Okubo H. (2006). Geographic distribution and habitat differentiation in diploid and triploid *Lilium lancifolium* of South Korea. J. Fac. Agric. Kyushu Univ..

[20] Truong N.X., Kim J.Y., Rai R., Kim J.H., Kim N.S., Wakana A. (2015). Karyotype analysis of Korean *Lilium maximowiczii* regal populations. J. Fac. Agric. Kyushu Univ..

[21] Truong N.X., Song Y.S., Kim N.S., Park J.W., Kim J.H., Wakana A. (2015). Occurrence and survival of autotriploids in natural diploid populations of *Lilium Lancifolium* Thunb.. J. Fac. Agric. Kyushu Univ..

[22] Asano Y. (1987). A numerical taxonomic study of the genus *Lilium* in Japan. J. Fac. Agric. Kyushu Univ..

[23] RHS (1966). Royal Horticultural Society (RHS) Color Chart.

[24] Mori T. (1921). An Enumeration of Plants Hitherto Known from Corea.

[25] KBIS (2021). Korea Biodiversity Information System. KBIS. http://www.nature.go.kr/eng.

[26] Kim Y.S., Shim K.M., Jung M.P., Choi I.T., Kang K.K. (2017). Study on the Change of Climate Zone in South Korea by the Climate Change Scenarios. Korean J. Agric. For. Meteorol..

[27] Willson E.H. (1925). The Lilies of Eastern Asia.

[28] Kosugi H. (2020). Lilium Leichtlinii and Sinomartagon in ‘Kingdom of Lilium’ 5.

[29] Inuma Y. (1856). Kooniyuri. Shohonzusestu. Kyoto.

[30] Burns J.H., Strauss S.Y. (2011). More closely related species are more ecologically similar in an experimental test. Proc. Natl. Acad. Sci. USA.

