# Supplementary material for: Morphological Variations between Korean and Southwestern Japanese Lilium leichtlinii Hook. f
Source: Plants (Basel). 2022 Aug 2;11(15):2016. doi: 10.3390/plants11152016 (PMC9370479; doi:10.3390/plants11152016)
Supplement: Supplementary file 1 [file plants-11-02016-s001.zip › plants-1811328-supplementary.pdf]

Supplementary Table S1. Matrix of coefficient vectors and variance proportion from the first three principal component axes based on 36 morphological traits of 59 *L. leichtlinii* accessions (30 KR and 29 JSW).

No	Trait*	Component and loading value			Uniqueness
		1	2	3	
1	3LL	**0.8381**	0.1947	0.25758	0.193
2	2LL	**0.8326**	0.1972	0.23716	0.212
3	1LW	**0.8095**	0.2510	0.00875	0.282
4	1LL	**0.7760**	0.2588	0.24302	0.272
5	4LL	**0.7741**	-0.0175	0.27212	0.326
6	3LW	**0.7730**	0.3418	0.15449	0.262
7	2LW	**0.7603**	0.3434	0.10012	0.294
8	5LL	**0.7272**	-0.0258	0.28183	0.391
9	4LW	**0.7037**	0.4147	0.19659	0.294
10	5LW	**0.6780**	0.4316	0.25778	0.287
11	LOB	0.6709	-0.0823	0.26730	0.472
12	WOB	0.6062	-0.4061	-0.13011	0.451
13	DOP	0.5134	-0.2341	-0.26229	0.613
14	LOBl	0.4804	0.4103	0.44514	0.403
15	LIFr	0.4363	0.2119	0.24370	0.705
16	LOT	0.1722	**0.8721**	0.22751	0.158
17	LIT	0.2026	**0.8707**	0.22509	0.150
18	DOSt	0.0739	**0.8345**	0.28196	0.219
19	LOF	-0.0961	**0.7216**	0.32563	0.364
20	WOT	0.1351	0.6616	-0.16343	0.517
21	WIT	0.2964	0.6440	0.13444	0.479
22	LOPI	0.1952	0.6186	0.43110	0.393
23	LOA	0.1522	-0.5998	-0.21583	0.571
24	MLW	0.0624	0.4810	0.47575	0.538
25	LOO	0.4004	0.4705	0.29697	0.530
26	LON	0.3705	0.4376	0.36736	0.536
27	NL	-0.1059	-0.2560	0.01765	0.923
28	NOP	-0.1779	-0.2506	0.24026	0.848
29	LT1F	0.2085	0.0525	**0.91692**	0.113
30	LTLL	0.2103	0.0221	**0.90631**	0.134
31	PLH	0.3415	0.1203	**0.85983**	0.130
32	LIN	0.1379	0.1772	**0.82674**	0.266
33	MLL	0.2734	0.2096	**0.74792**	0.322
34	NOBl	-0.2257	-0.3287	-0.55356	0.535
35	LPED	0.0776	0.3594	0.54578	0.567
36	LOP	0.2035	0.3374	0.45178	0.641
	Eigenvalue	13.92	4.62	3.06	
	% of variance	23.4	18.9	17.8	
	Cumulative %	23.4	42.2	60.0	

Note. Varimax rotation was used. *Abbreviations are shown in Table 2.
